# Non-Electroneutrality Generated by Bacteriorhodopsin-Incorporated Membranes Enhances the Conductivity of a Gelatin Memory Device

**DOI:** 10.3390/gels9080635

**Published:** 2023-08-07

**Authors:** U-Ting Chiu, Bo-Fan Lee, Ling-Ning Ko, Chii-Shen Yang, Ling Chao

**Affiliations:** 1 Department of Chemical Engineering, National Taiwan University, Taipei 10617, Taiwan; 2 Department of Biochemical Science and Technology, National Taiwan University, Taipei 10617, Taiwan

**Keywords:** bacteriorhodopsin, protein-bound water, gelatin memory device

## Abstract

We have previously demonstrated the potential of gelatin films as a memory device, offering a novel approach for writing, reading, and erasing through the manipulation of gelatin structure and bound water content. Here, we discovered that incorporating a bacteriorhodopsin (BR)–lipid membrane into the gelatin devices can further increase the electron conductivity of the polypeptide-bound water network and the ON/OFF ratio of the device by two folds. Our photocurrent measurements show that the BR incorporated in the membrane sandwiched in a gelatin device can generate a net proton flow from the counter side to the deposited side of the membrane. This leads to the establishment of non-electroneutrality on the gelatin films adjacent to the BR-incorporated membrane. Our Raman spectroscopy results show that BR proton pumping in the ON state gelatin device increases the bound water presence and promotes polypeptide unwinding compared to devices without BR. These findings suggest that the non-electroneutrality induced by BR proton pumping can increase the extent of polypeptide unwinding within the gelatin matrix, consequently trapping more bound water within the gelatin-bound water network. The resulting rise in hydrogen bonds could expand electron transfer routes, thereby enhancing the electron conductivity of the memory device in the ON state.

## 1. Introduction

Finding suitable materials to bridge electronic devices and biological systems is a crucial challenge in various biomedical applications [1,2,3,4]. Electronic devices rely on electron conduction, while biological systems operate in an aqueous environment that traditionally hinders efficient long-distance electron transfer. In our previous work, we demonstrated that gelatin hydrogels can function as an electron-conductive resistor by removing unbound free water while retaining bound water [5]. The formation of a protein-bound water network can facilitate long-distance electron transfer in gelatin films.

We used gelatin as the device material since it contains numerous sites capable of forming hydrogen bonds with water molecules [6]. Since previous studies have reported that hydrogen bonds can transfer electrons [7,8], it is possible that the bound-water-mediated hydrogen bonds can transfer electrons across different polypeptide chains. In a typical wet gel below the gelation temperature, bound water primarily resides within gelatin triple helices, limiting its ability to connect different helices. The dynamic free bulk water between different helices could also hinder the electron transfer and therefore causes low electron conductivity. The property to switch the electron conductivity based on the controllable structural change and bound water connection allows gelatin to have the ability to become a resistive memory.

Since the extent of polypeptide unwinding can significantly increase the electron conductivity and therefore the ON/OFF ratio of a gelatin resistive memory, it is important to investigate what factors can promote unwinding. Previous studies have demonstrated that the introduction of electrostatic repulsion can influence the winding and unwinding of biopolymers [9,10,11,12]. In this study, we incorporated bacteriorhodopsin (BR) into the sandwiched membrane in a gelatin memory device to induce non-electroneutrality under illumination, thereby promoting polypeptide unwinding. BR is a membrane protein that can pump protons under light illumination [13,14] and has been used for various photoelectrical applications [15,16,17,18,19]. When incorporated into a membrane, BR can transport protons across the membrane, generating spatial non-electroneutrality [20,21,22].

In this study, we discovered that the non-electroneutrality caused by BR proton pumping could promote the unwinding of the triple-helical structure and enhance the formation of protein-bound water, thereby facilitating electron transfer and creating a more conductive memory device. To demonstrate the relationship between BR proton pumping and conductivity, we conducted voltage sweep current–voltage (I–V) measurements on the gelatin devices. Our results clearly show that the conductivity of the devices in the ON state is positively correlated with the BR density ratio and that the presence of active proton pumping induced by light is essential for the observed conductivity enhancement. We also employed Raman spectroscopy to investigate the amount of bound water and the extent of polypeptide unwinding in the gelatin devices both with and without BR membranes. Our results suggested that the non-electroneutrality caused by BR pumping leads to an increase in bound water and unwound polypeptides, thereby significantly enhancing the conductivity of the gelatin devices.

## 2. Results and Discussion

### 2.1. Addition of BR Membranes to Gelatin Devices Significantly Enhances Electron Conductivity

**Figure 1:**
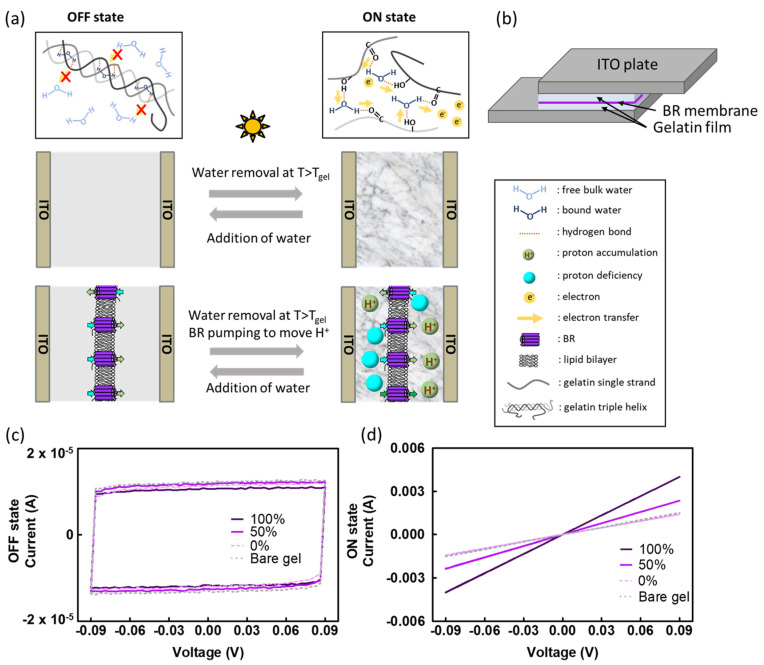
(**a**) Schematic representation of the proposed writing and erasing mechanism for the BR-incorporated gelatin memory device compared to the bare gelatin memory device. (**b**) Device setup. BR membranes were deposited on an ITO plate with a gelatin film, which was then sandwiched with another ITO plate with a gelatin film. (**c**) Voltage sweeps (0.36 V/s) depicting the capacitor behavior in the OFF state of the BR-incorporated gelatin memory device with varying BR density ratios and of the bare gelatin memory device. (**d**) Voltage sweep illustrating the resistor behavior in the ON state of the BR-incorporated gelatin memory device with different BR density ratios and of the bare gelatin memory device.

We prepared BR-incorporated gelatin devices with varying BR density ratios of 100%, 50%, and 0% (definitions in Materials and Methods). Figure 1a provides an overview of the device structure and the proposed mechanism for the writing process. Initially, the gelatin hydrogel possesses a triple-helical structure surrounded by free water molecules, acting as an insulator for electron transport. During the writing process, the temperature increases above the gelling temperature, causing the gelatin triple helices to unwind into single strands. As water content decreases, the gelatin single strands can compact together along with bound water molecules trapped within the matrix. Simultaneously, BRs actively pump protons across the membrane under illumination, resulting in an increase in protons on one side of the gelatin and a decrease on the other side. We propose that the alteration of the proton gradient by BRs further disrupts the gelatin secondary structure, allowing more amino acids in the gelatin polypeptides capable of forming hydrogen bonds to interact with bound water. The increased hydrogen bonds facilitated by bound water among the gelatin single strands provide additional routes for electron transfer within the protein-bound water network. Consequently, the BR-incorporated gelatin memory device could exhibit enhanced conductivity in the ON state.

To fabricate the BR-incorporated device, we deposited a BR membrane on an ITO plate with a gelatin film, which was then sandwiched with another ITO plate with a gelatin film, as illustrated in Figure 1b. The gelatin film on each of the ITO plates had a thickness of 152 μm, and the dried gelatin film after the water loss had a thickness of about 100 μm. The protruding ITO plates on both sides were clamped with alligator clips for subsequent electrical measurements. Due to possible preferential orientation during deposition, the gelatin surfaces on the deposition side and the counter side may experience different environments. More information can be found in the next subsection. 

Resistive memory devices store and access information by encoding “0” and “1” based on the electrical bistability of the active layer. Achieving a high current ratio of the ON state to the OFF state is often desirable [23,24]. Figure 1b,c show the electrical memory characteristics of the BR-incorporated gelatin memory device. The voltage sweep measurement, ranging from −0.09 to +0.09 V at a sweep rate of 0.36 V/s, was used to record the current. Figure 1b shows that the typical BR-incorporated gelatin hydrogel behaves as an ideal capacitor in the OFF state, similar to bare gelatin gel. Following the illumination writing process, the current of the 100% BR ratio sample increases to 4.0 × 10^−3^ A at 0.09 V, and it acts as a resistor in the ON state. The ON/OFF current ratio reaches 1.6 × 10^5^, which is two orders of magnitude higher than those in previous studies using bio-related materials for constructing memory devices [25,26]. Table 1 displays the conductivity of BR-incorporated gelatin hydrogel with different BR density ratios in the ON state. Increasing the density ratio of BR leads to enhanced conductivity. The 100% BR ratio sample exhibits a conductivity of 6.9 × 10^−5^ S/cm (n = 3), which is in the range of the conductivity of a semiconductor or electrically conductive polymers [27]. One-way analysis of variance (ANOVA) with a *p*-value of 0.012 demonstrates statistically significant differences in electron conductivity among the 100%, 50%, and 0% BR ratio samples. Notably, incorporating BR into a gelatin device increases the ON/OFF current ratio by approximately 2-fold, which is likely due to the additional protons and proton vacancies introduced during the writing process.

**Figure 2:**
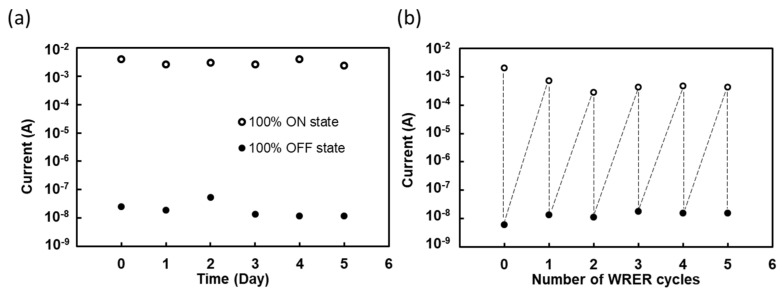
Memory characteristics of gelatin memory devices with 100% BR density ratio. (**a**) Retention time curves. (**b**) Switching stability.

This type of memory also demands repeatable switching between the ON and OFF states, and it often relies on voltage or current for the reprogrammability [24,28]. To assess the stability and reprogrammability of the BR-incorporated gelatin device, we conducted tests for retention time and WRER (write–read–erase–reread) cycles, as depicted in Figure 2a and Figure 2b, respectively. Figure 2a demonstrates that at 0.09 V, the increased current of the 100% BR-incorporated gelatin device can be maintained in the ON state for up to 5 days. Furthermore, Figure 2b reveals that the device is capable of enduring five WRER cycles, signifying its potential application in non-volatile memories.

### 2.2. Net Proton Flow toward the Deposited Side of the BR-Incorporated Membrane Revealed by Photocurrent Measurements

We hypothesized that the enhanced performance of the gelatin device can be attributed to the non-electroneutrality in the gelatin films caused by BR proton pumping. During the writing process, if the BRs in the sandwiched membrane can provide a net proton flow toward either the deposition side or the counter side of the gelatin films, the imbalanced proton concentrations can give rise to non-electroneutral states in both gelatin films.

**Figure 3:**
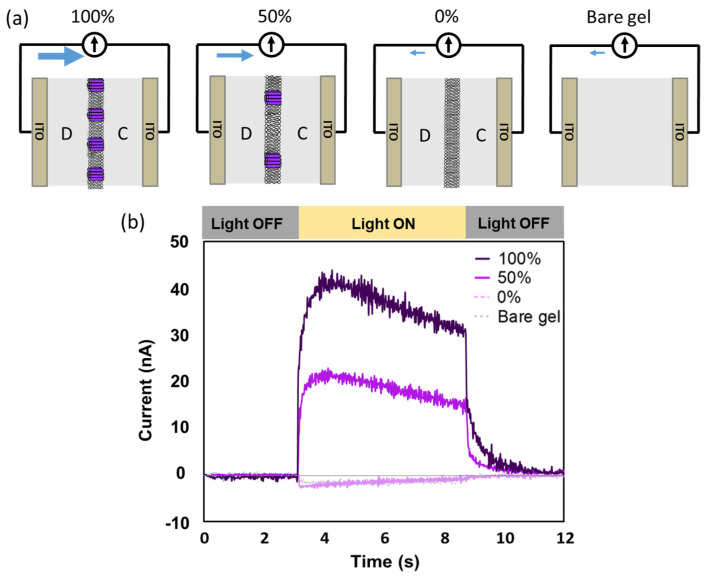
(**a**) Schematic representation of the gelatin devices in the OFF state used for the photocurrent measurements. D: Gelatin film at the deposited side of the sandwiched membrane. C: Gelatin film at the counter side. Blue arrows indicate the direction of photocurrent. (**b**) Photocurrent measurements obtained from the four different gelatin devices depicted in (**a**).

To determine the direction of the proton flow during the writing process, photocurrent measurements were conducted in the OFF state gelatin devices which still have abundant free bulk water for BRs to properly function. The presence of a photocurrent would indicate the existence of a net proton flow, with the direction of the photocurrent indicating the direction of proton movement [29,30]. Figure 3 illustrates the photocurrent results obtained from BR-incorporated devices with two different density ratios of BR (100% and 50%) as well as a device containing only a DOPC membrane without BR (0%). The device geometries are depicted in Figure 3a. During the illumination period of 3 to 8 s for the BR-incorporated gelatin devices, photocurrents were observed flowing from the deposited side electrode to the counter side in the external circuit (Figure 3b). The magnitude of the photocurrent increased with the BR density ratio in the membrane. These findings suggest that the BR-incorporated membrane, positioned in the middle of the device, facilitates a net proton flow from the counter side to the deposited side of the gelatin film.

Conversely, in the bare gelatin device and the 0% BR device, small negative currents were observed. The negative currents are likely attributed to the delocalization of electrons of the ITO plates, which is induced by the ultraviolet band in the light [31]. Since the system was illuminated from the deposited side, the ITO at the deposited side had a higher likelihood of electron delocalization and pressure compared to the ITO plate at the counter side. As a result, electron movement occurred from the deposited side electrode to the counter side electrode in the external circuit, which is equivalent to a current from the counter side electrode to the deposited side electrode in the external circuit. 

It is important to note that the physical state of the BR-incorporated membrane within the gelatin device has not been elucidated. Whether the prepared liposomes rupture to form supported lipid bilayers or crumple on the gelatin film remains unclear. Additionally, the specific orientation of BR within the membrane is also unclear. Nevertheless, what we can affirm is the presence of an overall net proton flow from the counter side to the deposition side of the gelatin device, leading to non-electroneutrality on both sides of the gelatin films.

### 2.3. The Proton Pumping Enhances the Conductivity of the Gelatin Device in the ON State

**Figure 4:**
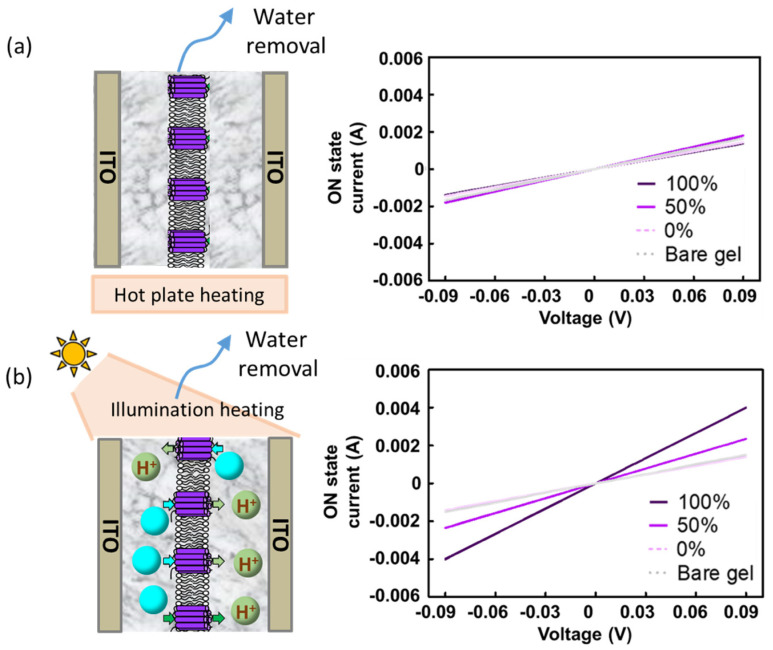
(**a**) Illustration and voltage sweep results of the gelatin device in the ON state using hotplate heating at 60 °C for 1 h as the writing process. (**b**) Illustration and voltage sweep results of the gelatin device in the ON state using illumination as the writing process.

To further investigate how the incorporation of BRs increases electron conductivity, we employed two different heating methods for the writing processes: hotplate heating and illumination heating. During hotplate heating, BRs remain inactive, since they require light absorption to pump protons. In contrast, during illumination heating, BRs contribute additional protons and proton vacancies to the writing process. The voltage sweep curve in Figure 4a demonstrates that the conductivity values for all devices were nearly identical when hotplate heating was used, indicating that inactive BRs did not significantly contribute to the conductivity. On the other hand, when the illumination writing process was applied, the conductivities increased with the BR density ratio in the membrane (Figure 4b). These findings support the notion that the increase in conductivity in the ON state is primarily attributed to the proton pumping activity of BRs rather than the mere presence of the BR material.

### 2.4. The Increased Conductivity Is Not Due to the pH Change

**Figure 5:**
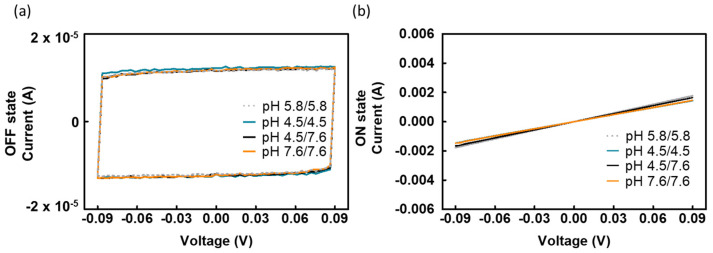
Voltage sweeps demonstrating (**a**) similar capacitor behaviors of gelatin films at various pH values in the OFF state, and (**b**) comparable resistor behaviors of gelatin films at various pH values in the ON state.

As BR proton pumping can potentially alter the pH across the membrane [29,30], we investigated whether the increased conductivity was a result of pH change. We conducted experiments using gelatin films prepared at pH = 4.5 and pH = 7.6 to simulate varying pH values on either side of the BR membrane. We chose these two pH conditions since one was more acidic and the other was more basic than our typical working condition at pH = 5.8. Figure 5a,b display the electrical characteristics of these gelatin films in the OFF and ON states. The conductivities of the gelatin films at different pH values were found to be similar and are summarized in Table 2. It is apparent that the conductivity values of the gelatin films at pH = 4.5, 7.6, and the combination of pH 4.5 and 7.6 were comparable to that of the gelatin films at pH = 5.8. None of these conditions can elevate the conductivity to the level achieved by BR-incorporated gelatin films. These findings strongly suggest that the improved conductivity is not attributable to variations in the acidic or basic conditions.

### 2.5. Raman Spectroscopy Analysis Shows Increased Amounts of Bound Water and a More Disordered Gelatin Structure in the ON State Gelatin Devices

To investigate whether the presence of BR proton pumping activity could enhance the bound water content in the ON state gelatin devices, the Raman spectra of gelatin devices at pH = 4.5, 5.8, and 7.6 without BR membranes were compared with that with BR membranes. The peaks observed at 3320 cm^−1^ and 3450 cm^−1^ correspond to the OH stretching vibrations of tightly bound water [32,33,34], which can be utilized to quantify the amount of bound water. The peaks in the range of 2800–3000 cm^−1^ originate from the CH stretching of gelatin. To ensure comparability, all spectra were scaled to achieve the same intensity of the CH stretching, thereby allowing for the normalization of bound water OH-stretching intensities by the gelatin content.

**Figure 6:**
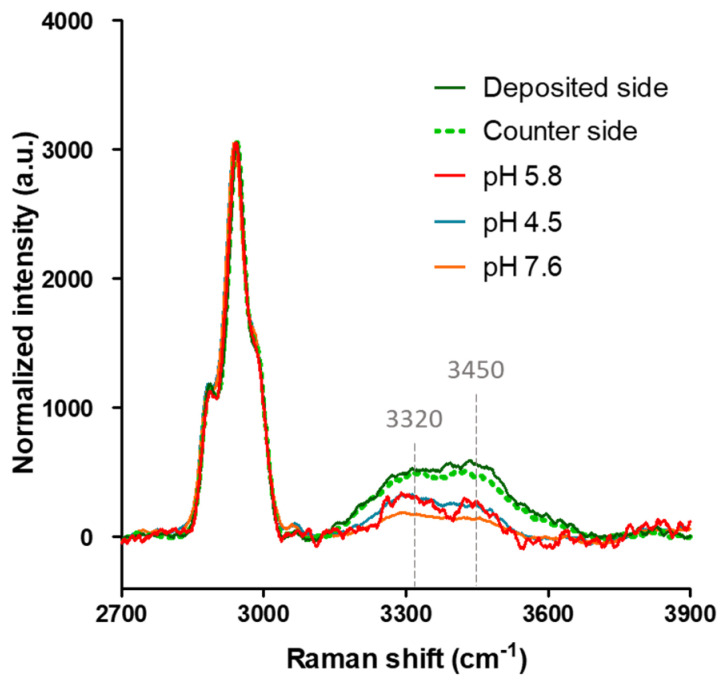
Comparison of Raman spectra from gelatin films at various pH values and on different sides of the BR-incorporated gelatin device in the ON state. The peaks at 3320 cm^−1^ and 3450 cm^−1^ correspond to the OH stretching vibrations of tightly bound water.

Figure 6 presents the Raman spectra of the gelatin films in the BR-incorporated gelatin devices, either the deposited side or the counter side, and they exhibited higher bound water contents compared to the devices without BR membranes at various pH values. There were no significant differences in the bound water contents among the gelatin devices with different pH values. The higher bound water content suggests that the gelatin polypeptides were in a more unwound state, thereby providing more amino acids capable of forming hydrogen bonds with water molecules. The presence of additional water-mediated hydrogen bonds within the protein-bound water network could facilitate electron transfer, resulting in enhanced conductivity. These findings align with the increased conductivity observed through BR proton pumping, as indicated in Table 1. 

Raman spectra covering the protein amide region were also examined to study how the protein secondary structure is influenced by the BR proton pumping. Previous studies have indicated that 1530–1650 cm^−1^ range in the Amide I spectrum region corresponds to the ordered secondary structure. A decrease in the peak intensity suggests the increased disorder of a protein structure [35,36,37]. In addition, the intensity reduction at 1268 cm^−1^ in the Amide III spectrum region indicates the loss of the triple-helix structure [38,39]. 

**Figure 7:**
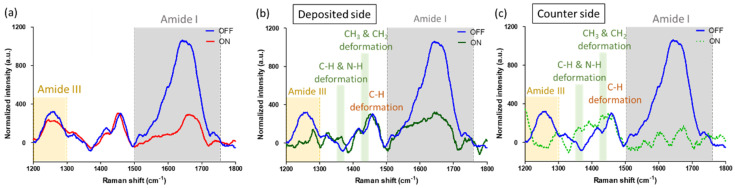
Raman spectra in the amide regions: (**a**) Gelatin device without BR membranes; (**b**) Gelatin device with BR membranes at the deposited side; (**c**) Gelatin device with BR membranes at the counter side.

Figure 7a depicts the Raman spectra of a representative gelatin device without BR membranes. Comparing the ON state with the OFF state, lower intensities at 1530–1650 cm^−1^ and 1268 cm^−1^ suggest a loss of the triple-helix structure [38,39]. Figure 7b,c present the Raman spectra of the gelatin films at the deposited and counter sides of a 100% BR ratio memory device. In both cases, the spectra in the ON state exhibit significantly reduced intensities at 1530–1650 cm^−1^ and 1268 cm^−1^ compared to the OFF state spectra, indicating the loss of secondary structure. Notably, the decreases in the Raman intensities in the ON state BR-incorporated devices are more pronounced than those in the ON state gelatin device with no BR membranes, suggesting a more complete loss of secondary structure in the presence of BR membranes. Furthermore, an increase in intensity within the 1350–1460 cm^−1^ range, corresponding to CH, CH2, and CH3 vibrations, was observed in the ON state BR-incorporated gelatin device compared to the OFF state, implying greater flexibility in the gelatin polypeptide backbones. This increased flexibility supports the notion of more extensive unwinding of the gelatin polypeptides in the ON state BR-incorporated devices.

### 2.6. Proposed Mechanism of How Non-Electroneutrality Influences the Gelatin-Bound Water Network in the ON State

**Figure 8:**
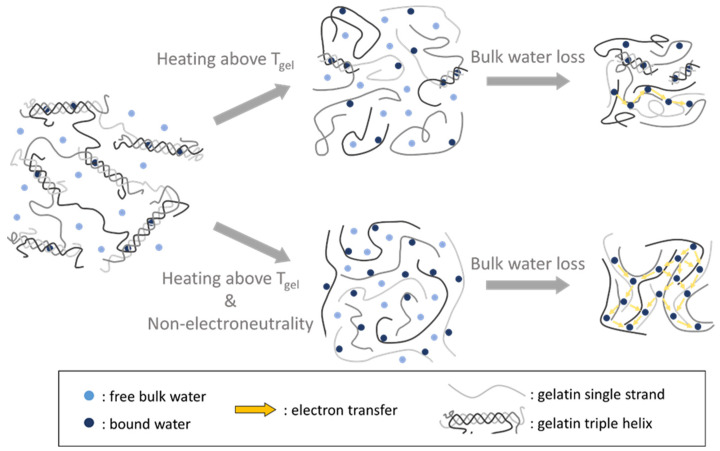
Illustration of the effect of non-electroneutrality on gelatin structure after the writing process. During the writing process, non-electroneutrality induces a more thorough unwinding of the triple-helical structure into single polypeptide strands. This unwinding exposes a greater number of hydrogen-bonding sites on the polypeptides, facilitating the formation of hydrogen bonds with bound water molecules. As a result, the gelatin-bound water network exhibits a higher bound water content, enabling multiple pathways for electron transport, which in turn leads to an increase in device conductivity.

Based on the electrical performance measurements and the Raman spectra obtained under various conditions, we propose a mechanism (Figure 8) in which the non-electroneutrality induced by BR proton pumping enhances the conductivity of a gelatin device in the ON state. During the illumination writing process, proton pumping across the membrane leads to non-electroneutrality on both sides of the gelatin films. This non-electroneutrality could induce electrostatic repulsion between individual gelatin polypeptide strands, promoting the unwinding of the triple-helical structure. This structural change, facilitated by non-electroneutrality, likely prevents the self-association of gelatin single strands as the triple helices unwind. With more unwound strands, more bound water molecules can trap and stay in the gelatin matrix during the writing process. This process allows more routes for electron transport in the gelatin-bound water network, and therefore, the conductivity increases. 

## 3. Conclusions

Our study revealed that the incorporation of the proton-pumping protein bacteriorhodopsin (BR) through an illumination writing process has a significant impact on the gelatin-bound water network, leading to enhanced conductivity in the gelatin memory device. We demonstrated that the conductivity in the ON state can be further increased by augmenting the BR density ratio in the membrane incorporated in a gelatin device. To establish the underlying mechanism, we employed different writing methods and created varying pH environments for the gelatin, enabling us to attribute the conductivity enhancement to the non-electroneutrality induced by BR proton pumping. Raman spectroscopy analyses supported our findings by indicating that BR proton pumping promotes the unwinding of the gelatin’s triple-helical structure. The more unwound gelatin polypeptide chains could be capable of forming additional hydrogen bonds with water molecules. This phenomenon allows for the trapping of a greater number of bound water molecules within the gelatin-bound water network, thereby enhancing the efficiency of electron transfer. Our findings provide valuable insights into the influence of non-electroneutrality on gelatin structure and its impact on the conductivity and performance of the memory devices. This research opens up promising opportunities for enhancing the functionality and conductivity of gelatin-based devices by using the non-electroneutrality effects induced by proton-pumping proteins such as BR.

## 4. Materials and Methods

### 4.1. BR-Incorporated Liposome Preparation

The HmBRI/D94N bacteriorhodopsin mutant was obtained from Professor Chii-Shen Yang’s group at National Taiwan University. To express HmBRI, the mutant plasmid was transformed into *E. coli* and allowed to undergo overexpression [40]. Subsequently, HmBRI was purified using octyl glucoside as a detergent. To incorporate HmBRI into liposomes, purified HmBRI was mixed with a 50 mg/mL solution of DOPC (1,2-dioleoyl-sn-glycero-3-phosphocholine) liposomes at a weight ratio of 1:100. The mixture was then dialyzed for 24 h at 4 °C to remove the octyl glucoside detergent, allowing HmBRI to insert into the DOPC liposomes. To obtain liposomes with various BR density ratios, a mixture of the prepared BR-incorporated DOPC liposomes and pure DOPC liposomes at a concentration of 50 mg/mL was prepared, using different volume ratios as required.

### 4.2. Preparation of Gelatin Solution

To prepare a 10 wt% gelatin solution, 1 g of Type B gelatin powder (Sigma-Aldrich, St. Louis, MO, USA) was added to 10 g of 100 mM NaCl solution (pH = 5.8). The mixture was gently stirred for 2 h at 60 °C to ensure complete dissolution and achieve a homogeneous gelatin solution.

### 4.3. Fabrication of BR-Incorporated Gelatin Memory Devices

First, 200 μL of 60 °C gelatin solution was placed on an ITO plate (7 Ω, 2 × 2.5 cm^2^ in size Ruilong-glass Co., Ltd., Bengbu, China) and immediately spincoated at 2000 rpm for 20 s at 25 °C. The gelatin thin films were then dried at 4 °C for 30 min. Subsequently, liposomes were added onto the gelatin films for 1 h, and then, the samples were washed with 100 mL of 100 mM NaCl solution (pH 5.8). To fabricate the BR-incorporated gelatin memory device, the gelatin-coated ITO plate with the deposited BR membrane was sandwiched with another gelatin-coated ITO plate.

### 4.4. Electrical Performance Measurements

Electrical performance measurements were conducted using a Keithley 2636B sourcemeter from TEKTRONIX, Inc. The voltage sweep current–voltage (I–V) characteristics were recorded under a sweep rate of 0.36 V/s without light illumination. For the measurement of current–time characteristics, the gelatin-based devices were illuminated using a Xenon lamp system (Prosper OptoElectronic Co., Ltd., Yilan, Taiwan) of AM1.5. The stability performance of the gelatin-based memory device was tested at a reading voltage of +0.09 V. Prior to the reading measurements, the devices were illuminated for 1 h during the writing process and then cooled to ambient temperature. The erasing process was accomplished by immersing the devices in a 100 mM NaCl solution (pH = 5.8) for 1 h. All measurements were performed under environmental conditions with a relative humidity of 40%.

### 4.5. Raman Spectroscopy to Examine the Bound Water Content and Gelatin Secondary Structure

Raman measurements were carried out using the inVia™ confocal Raman microscope (Renishaw, Wotton-under-Edge, UK) equipped with a He-Ne laser for 633 nm excitation. The average laser power was set at 14.1 mW. Renishaw WiRE 5.0 software was used for data retrieval and analysis. All Raman spectra, including the gelatin and water OH-stretch regions, were normalized to have the same intensity for the CH-stretch band at 2950 cm^−1^, representing the gelatin content in the samples. Additionally, the spectra containing the amide band were normalized to obtain the same intensity for the CH_2_ wag band at 1450 cm^−1^. All spectra were subjected to smoothing and baseline subtraction to eliminate the background signal.

## Figures and Tables

**Table 1:** Conductivity of BR-incorporated gelatin devices with varying BR density ratios (n = 3).

BR Density Ratio in the DOPC Membrane	OFF State Capacitance (μF/cm^2^)	ON State Conductivity (×10^−5^ S/cm)
100%	41 ± 2	6.9 ± 0.9
50%	42 ± 1	4.9 ± 0.3
0%	43.2 ± 9.1	2.7 ± 0.5
Bare gel (no membrane)	42.2 ± 3.2	3.4 ± 0.6

**Table 2:** Conductivity of gelatin films at various pH values (n = 3).

pH Value	ON State Conductivity (×10^−5^ S/cm)
5.8/5.8	3.4 ± 0.6
4.5/4.5	3.2 ± 0.5
4.5/7.6	3.7 ± 0.7
7.6/7.6	3.3 ± 0.6

## Data Availability

The data presented in this study are available on request from the corresponding author.
